# Gastroprotective Effect of Microencapsulated *Myrtus communis* Essential Oil against Ethanol/HCl-Induced Acute Gastric Lesions

**DOI:** 10.3390/molecules27051566

**Published:** 2022-02-26

**Authors:** Rim Ben Mansour, Raja Serairi Beji, Hanen Wasli, Sami Zekri, Riadh Ksouri, Wided Megdiche-Ksouri, Susana M. Cardoso

**Affiliations:** 1 Laboratory of Aromatic and Medicinal Plants, Centre of Biotechnology of Borj-Cedria, BP 901, Hammam-Lif 2050, Tunisia; rim.bmansour@gmail.com (R.B.M.); raja_serairi@yahoo.fr (R.S.B.); hanenwasli@gmail.com (H.W.); ksouririadh@gmail.com (R.K.); ksouriwided@yahoo.fr (W.M.-K.); 2 High School of Health Sciences and Technics, BP 176, Bab Saadoun, Tunis 1007, Tunisia; 3 USCR, Unité de Services Communs pour la Recherche en Microscopie Electronique à Transmission, Faculté de Médecine de Tunis, Tunis 1029, Tunisia; semy66@gmail.com; 4 LAQV-REQUIMTE & Department of Chemistry, University of Aveiro, 3810-193 Aveiro, Portugal

**Keywords:** *Myrtus communis*, essential oil, microencapsulation, spray drying, gastroprotective activity, anti-inflammatory

## Abstract

*Myrtus communis* L. essential oil (EO), mainly composed of myrtenyl acetate (30.6%), linalool (14.9%), α-pinene (11.10%) and 1,8-cineole or eucalyptol (9.9%), was microencapsulated with maltodextrin by emulsification and spray-drying, reaching a yield and efficiency of 43.7 and 48.7%, respectively. The microencapsulated myrtle EO (MMEO) was then evaluated regarding its gastroprotective activity in a model of ethanol/HCl-induced acute gastric ulcer in Wistar rats. Pretreatment with MMEO induced a remarkable inhibition of gastric lesions and acidity, correlated to high healing and protection percentages. Moreover, it exerted a potent anti-inflammatory effect on the gastric mucosa, counteracting EtOH-induced gastric lipoperoxidation and preventing the depletion of the antioxidant enzyme activity of superoxide dismutase (SOD), catalase (CAT) and glutathione peroxidase (GPx). Taken together, the gastroprotective action of encapsulated MMEO may be multi-factorial, and ascribable, at least in parts, to its anti-inflammatory and antioxidant properties.

## 1. Introduction

Gastric ulcers are part of a chronic, recurring disease, affecting a high number of people in the world and considered the new ‘‘plague of the 21st century’’ [1]. Currently, there is a significative amount of scientific data demonstrating that ulcers are often correlated with lipid peroxidation and oxidative damage of the mucosa, which is ultimately due to the oxidative damage of biological macromolecules such as DNA, protein and lipids occurring under oxidative stress conditions [2].

Conventional treatments of gastric ulcers based on pharmaceutical drugs such as omeprazole and ranitidine may elicit adverse complications such as hypersensitivity, arrhythmia, impotence and hematopoietic alterations [3]. Moreover, regarding the risk of developing gastric cancer, there may be a relationship between time and dose-dependence of using this type of medicine [4]. These data emphasize the need to search alternative treatment methods, through the screening of natural products, as prominent sources for the development of new agents with a safe therapeutic window.

According to the World Health Organization (WHO) [5], plant bioactive substances are a safe alternative to finding new therapies for the treatment of gastric ulcer due to their accessibility, efficacy and potentially fewer side effects. In this context, the protective and curative actions of several monoterpenic compounds such as carvacrol [6], citronellol [7], α-pinene [8], myrtenol [9], α-terpineol [10], eucalyptol [11], limonene [12,13], thymol [14], and β-myrcene [15] have been previously screened in experimental models of gastric ulceration. Related to this, several authors have evaluated the potential of essential oils (EOs) and of their derived compounds, aiming at the development of new gastro-protective drugs [16,17].

Regardless of the benefits, EOs, as well as monoterpenes, generally exhibit low water solubility, short plasma half-life and high volatility [18]. In this context, microencapsulation systems have emerged as a potential technological strategy to counter these constraints. In fact, distinct studies have previously reported a positive effect of incorporating essential oil in different wall materials (e.g., maltodextrin, cyclodextrin or gelatin) on the biological properties of their constituents [19]. Spray-drying is the most used technique for the encapsulation of EOs due to its relative simplicity, speed, low production cost, and reproducibility, allowing easy scaling compared with other encapsulation techniques, justifying its preferential use in the industrial sector [20].

*Myrtus communis* L., belonging to the Myrtaceae family, is commonly used in traditional medicine as decoction to treat stomached and gastrointestinal disorders such as diarrhea, constipation and peptic ulcer [21]. Moreover, distinct studies have previously demonstrated the disinfectant, antiseptic, antimicrobial and antioxidant capacities of *M. communis* EO, as well as potentialities in fighting several diseases, including rheumatic, respiratory and bladder diseases [22,23]. In addition, a previous study highlighted the antiulcer capacity of orally administered methanolic extracts of *M. communis* leaves [24] and berries [25], in experimental models of gastric ulceration [26].

In this study, we evaluated the gastroprotective effect of microencapsulated myrtle essential oil (MMEO) against ethanol/hydrogen chloride (EtOH-HCl)-induced acute gastric lesions in rats.

## 2. Results and Discussion

### 2.1. GC-MS Analysis and Microcapsule

**Figure 1:**
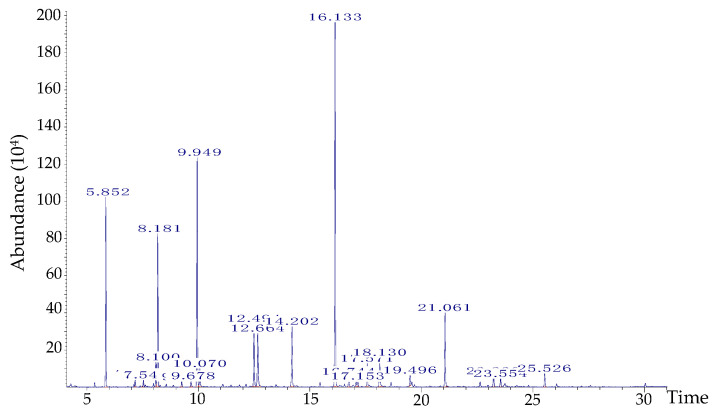
GC-MS chromatogram of *Myrtus communis* essential oil.

As shown in Figure 1 and Table 1, the main components of *M. communis* EO were oxygenated monoterpenes (55.4%), particularly the myrtenyl acetate (30.6%), linalool (14.9%) and 1,8-cineole or eucalyptol (9.9%). Monoterpene hydrocarbons were the second dominant components, mostly represented by α-pinene (11.1%) and limonene (1.63%). These results agree with the findings of Bazzali et al. [26], who have reported the presence of myrtenyl acetate, α-pinene and 1,8-cineole as the main constituents of Moroccan *M. communis* EO. In turn, other authors have reported that limonene, linalool, α-pinene, and 1,8-cineole (not myrtenyl acetate) are the main compounds in myrtle EO of Azerbaijan and Iranian origin, alerting us to the peculiarities of ecotypes [27,28].

Pure myrtus EO is not soluble in pure water at room temperature, whereas MMEO resulted in better solubility (results not shown). Maltodextrin was used as wall material for the production of MMEO microspheres, as a powdery form following spray-drying. A microencapsulation yield of 43.71% was obtained, which is a close value to those obtained for spray-dried microencapsulated rosemary EO using gum arabic/starch/maltodextrin/inulin as wall materials [29].

The microencapsulation efficiency (ME) analysis is one important quality parameter of EOs encapsulation and represents the percentage of entrapped EO in the capsule. The MMEO formulation presented a microencapsulation efficiency value of 48.76%. When evaluating the influence of several wall materials (gum arabic, starch, maltodextrin and inulin) on the ME of rosemary EO using spray-drying, Fernandes et al. [29] found ME values between 26.31 and 61.81%.

### 2.2. Effect of MMEO against Ethanol/HCl-Induced Gastric Ulcers

The administration of MMEO at 250, 500 and 1000 mg/kg for 21 days did not result in any sign of toxicity and mortality of the rats, since during that period of observation, there was no abnormal behavior among the animals in terms of salivation, diarrhea, hyper-excitability, respiratory suffering, and mortality, compared with the control group. Based on that, it was concluded that the three doses of MMEO were safe to be administered to the animals, in order to test and evaluate their gastroprotective effects.

**Figure 2:**
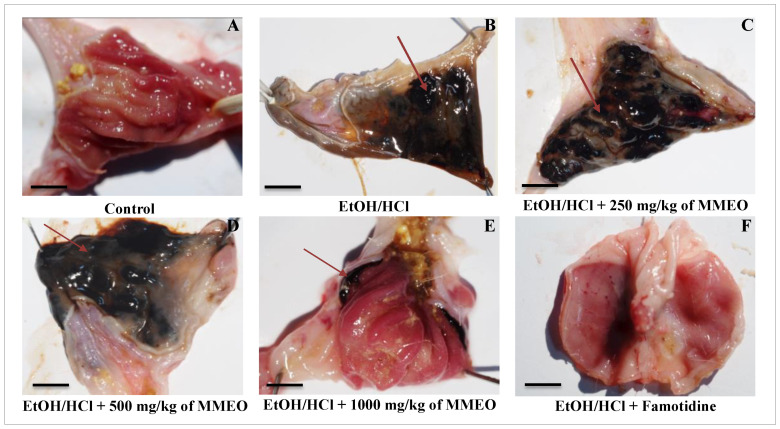
Photomicrographs showing mucosal surface of rat stomach, (**A**) stomach from control rat, (**B**) Stomach from rat treated with EtOH/HCl. Arrows indicate hemorrhage sites. (**C**–**E**) Stomach from rat treated with 250, 500 and 1000 mg/kg of MMEO, respectively + EtOH/HCl. (**F**) rat treated with 20 mg/Kg of famotidine + EtOH/HCl. Bar: 1 cm.

Both macroscopic and microscopic parameters are important tools in understanding the cellular processes in the EtOH/HCl-induced ulcer model. Representative images of the gross appearance of the gastric mucosa are shown in Figure 2. Severe tissue damage and visible hemorrhagic mucosal black or reddish-brown lesions and a distinctive “gelatin” aspect were found in the stomachs of the EtOH/HCl-treated group (i.e., ulcerated group), contrasting with the control group (i.e., received physiological saline solution) that exhibited no lesions in the gastric mucosa.

The MMEO treatment minimized the pathologic changes induced by EtOH/HCl, reducing inflammatory cell infiltration and submucosal edema in a dose-dependent manner. In particular, the administration of MMEO at 500 mg/kg significantly ameliorated the gastric tissue, allowing it to maintain its normal structure. Notably, the protective effect of MMEO at 1000 mg/kg was similar to that afforded by the pharmaceutical drug famotidine.

**Figure 3:**
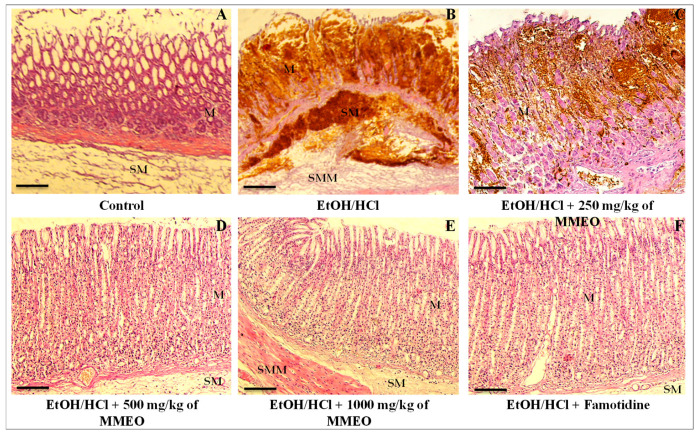
Histological findings of rat gastric mucosa. (**A**) stomach from control rat. (**B**) Stomach from rat treated with EtOH/HCl. Exacerbation of necrosis of surface mucus cells and glands, hemorrhage in mucosal layer and edema of submucosal layer were observed. (**C**–**E**) Stomach from rat treated with 250, 500 and 1000 mg/kg of MMEO, respectively + EtOH/HCl. Dose dependent reduced injuries were observed. (**F**) Rat treated with 20 mg/Kg of famotidine + EtOH/HCl. No obvious injuries are recognized in mucosal layer. Bar: 100 µm. M: mucosal layer; SM: submucosal layer.

Microscopic observations of control animals’ gastric mucosa showed a normal aspect (Figure 3A). On the other side, the gastric mucosa of EtOH/HCl-treated group (Figure 3B) manifested broad lesions such as necrosis of surface mucous cells, edema and hemorrhages, and polynuclear infiltration. These results suggest that initial inflammation and migration of activated leukocytes into the necrotic areas of mucosa occurred within 4 h. Treatment with MMEO minimized the degeneration of mucous cells induced by EtOH/HCl, inflammatory cell infiltration and submucosal edema in a dose-dependent manner. Gastric mucosa of rats treated with 1000 mg/Kg of MMEO was almost normal in appearance (Figure 3E). Necrosis of surface mucous cells was rarely observed and if noted, was not severe. This is consistent with the general knowledge that the oral gavage of absolute ethanol in rodents is toxic for the stomach, distressing the gastric mucosa topically by upsetting its barrier and inciting prominent micro vascular changes within a few minutes of its application. Thus, rapid and strong vasoconstriction is accompanied by vigorous arteriolar dilation, and this combination of micro-vascular events stimulates damage in mucosal capillaries [30,31].

Compared with the control group, gastric lesions produced by EtOH/HCl resulted in inflammation of the mucosa and the formation of several ulcers (ulcer index, UI = 2.66) and hemorrhagic furrows (% of ulceration = 85.16%) concomitant with a decrease in gastric juice volume from 4.26 to 1.75 mL, and of pH from 5.09 to 2.04 (Table 2). In turn, the oral pre-treatment with 250, 500 and 1000 mg/kg of MMEO ameliorated the gastric parameters, including re-establishment of mucosa, gastric pH and volume. In particular, administration of a high MMEO dose (1000 mg/kg, *p* < 0.001) set the index and the percentage of ulceration respectively at 1.63 and 19.66% and conferred a percentage of protection (PP) and healing (HP) of 83.33% and 69.97%, respectively, plus a sharp restoring of acidity and pH. This is consistent with previous reports demonstrating the gastroprotective and ulcer healing properties of EOs through reduced gastric volume and acidity, decreased lipid peroxidation, and increased mucus production [13,32].

It is feasible to hypothesize that the protective and curative actions of MMEO which succeeded in raising the pH level and reducing the acidity and lesion counts in stomach ulcerative rats are closely related with its main components. According to the bibliography, α-pinene (3rd major compound in MMEO) is widely accepted to exert gastroprotective activity, causing a significant inhibition of gastric mucosal lesions induced by ethanol, which might be associated, at least in part, with an increase of mucus secretion and reduction of gastric H^+^ secretion [8]. More recently, Rocha et al. [33] reported that α-pinene (30 mg/kg) reduced up to 44% of the gastric lesions induced by ethanol. Moreover, (-)-linalool administered orally at doses of 5, 10, 20 or 40 mg/kg [34], (-)-myrtenol at oral doses of 25, 50 and 100 mg/kg [10] and α-terpineol at the doses of 10, 30, and 50 mg/kg [10], were demonstrated to significantly decrease the severity of ethanol-induced ulcer, affording gastroprotection.

**Figure 4:**
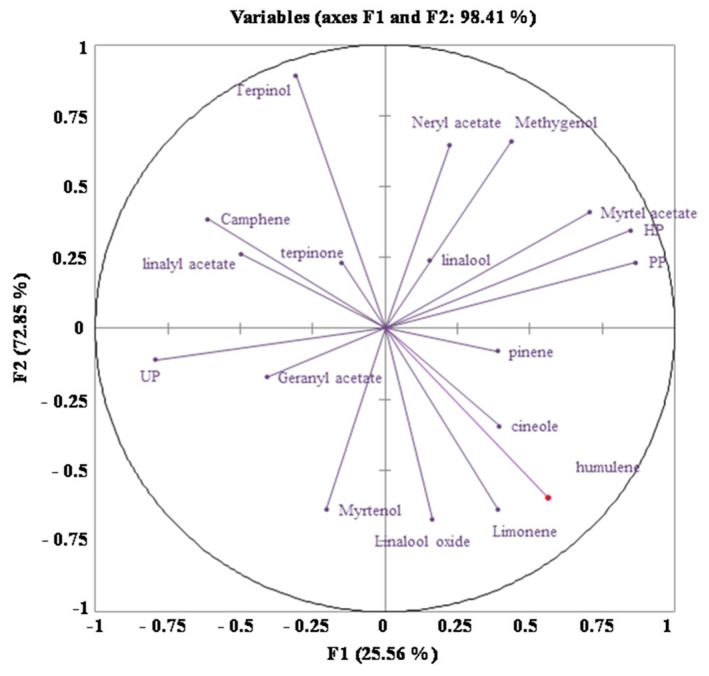
Principal component analysis (PCA) of the oral pre-treatment with microencapsulated *M. communis* essential oil (MMEO) showing the correlation between essential oil components and anti-inflammatory parameters. The first two components (PCs) contributed 98.41% to cumulative variance, with PC1 (F1 axis) and PC2 (F2 axis) explaining 72.85 and 25.56% of the total variance.

To further understand the contribution of MMEO constituents on the EO gastroprotective abilities, the direct relation between the protection, healing, and ulceration percentage with the concentration of the involved volatile components in MMEO and Pearson’s correlation coefficients analysis were evaluated (Figure 4 and Table 3, respectively). Overall, the results showed a strong contribution of α-pinene (r = 0.68; r = 0.60; r = 0.52), myrtenyl acetate (r = 0.73; r = 0.59; r = 0.92), geranyl acetate (r = 0.55; r = 0.66; r = 0.69) and methyl eugenol (r = 0.60; r = 0.51; r = 0.55) with the gastroprotective effect, which authenticate their role in scraping ulcer disease. These findings demonstrate that the gastroprotection of MMEO appears to be related to its multi-factorial actions.

### 2.3. Effect of MMEO on Nitric Oxide Levels

**Figure 5:**
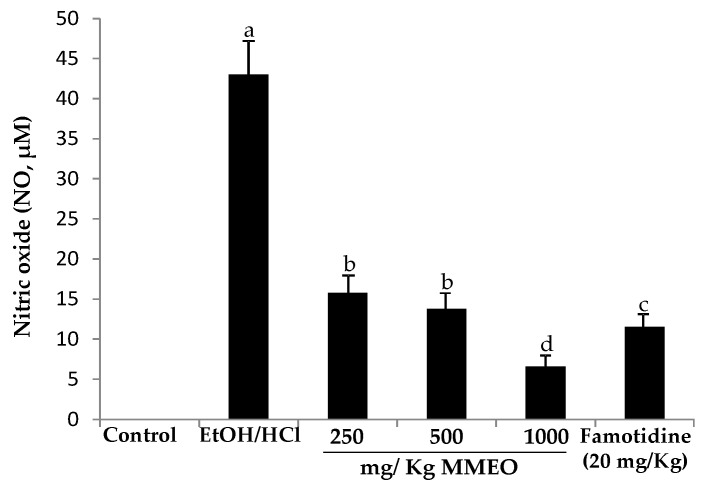
The effect of oral pre-treatment of ulcerated rat with microencapsulated *M. communis* essential oil (250, 500 and 1000 mg/kg MMEO) and famotidine, on nitric oxide (NO) production in gastric homogenate. Values are the means of three replicates and standard deviation. Values with different superscripts (a, b, c and d) are significantly different at *p* < 0.05.

The levels of nitric oxide in the mucosa homogenate were measured. As shown in Figure 5, the production of NO was much higher in the ethanol/HCl-induced ulcer control group (43 µM/mL) as compared with the normal control group, which only received saline solution. Meanwhile, famotidine and MMEO at all tested doses caused a sharp decrease on the NO levels.

It has previously been reported that one of the pathogeneses of ethanol-induced ulcer is the elevation of NO synthesis [35,36]. Interestingly, the prophylactic activity of MMEO not only diminished ethanol/HCl induced gastric injury (Figure 5) but also led to a significant reduction of inflammation in the mucosa (Figure 2). The capacity of MMEO to attenuate the inflammatory response may represent a key factor in the anti-ulcer potential of this natural remedy.

Several studies have demonstrated the positive effect of plant EOs through modulation of inflammatory mediators, antisecretory and anti-oxidative stress defense. For example, the study of Arunachalam et al. [37] showed that acidified ethanol and piroxicam as well as ulcer healing on acetic acid-induced ulcer models in rodents provoked the secretion of a high amount of NO in gastric explants; whereas oral pretreatment with *Gallesia integrifolia* EO exerted significant prophylactic and therapeutic effects against gastric ulcers traduced by a significant decrease of NO production. Along with that, different authors have reported that eucalyptol can be used as an anti-inflammatory agent to control mucus hypersecretion, which acts in the upkeep of gastric microcirculation [11,38]. Moreover, according to Viana et al. [9], the potent anti-inflammatory effect of myrtenol could be explained by its related structure to α-pinene. Indeed, Azab et al., have reported that a high percent of 1,8-cineole in *Cinnamomum glanduliferum* EO protected against gastric lesions and gastritis induced by ethanol administration in rats by reducing NO and malondialdehyde (MDA) levels in the gastric homogenate [16]. Furthermore, α-pinene (50.8%) and cineole (20.3%), major components of *Hyptis spicigera* EO, displayed antiulcerogenic and gastroprotective actions in gastric mucus production [39].

In order to associate the role of terpenoid compounds and to determine the key components involved in anti-inflammatory mechanism, we exploited the correlation relation between NO^●^ production inhibitory activities with the myrtle EO constituents. Curiously, a strong correlation was observed for myrtenyl acetate (r = 0.89), providing evidence for the efficient role of myrtenyl acetate in neutralizing nitric oxide radicals, while a weak/moderate link was established for α-pinene, geranyl acetate and methyleugenol.

### 2.4. Effect of MMEO on Lipid Peroxidation and Antioxidant Enzymes

**Figure 6:**
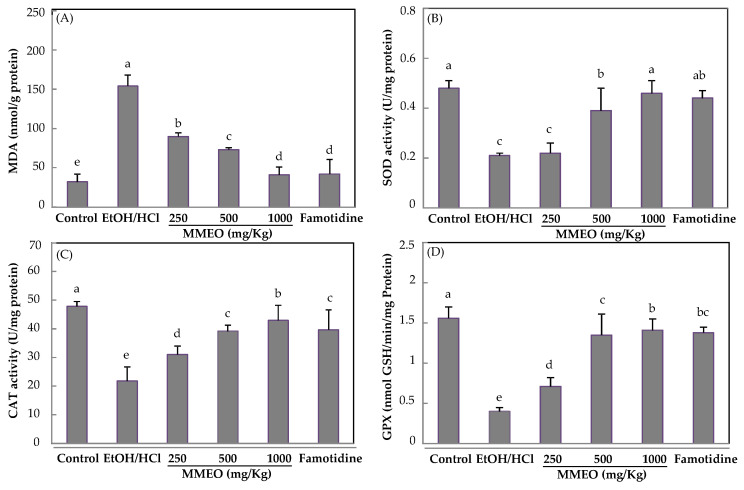
Effect of oral pre-treatment with microencapsulated *M. communis* essential oil (MMEO, 250, 500 and 1000 mg/Kg) on MDA concentration (**A**), and superoxide dismutase (SOD) (**B**), catalase (CAT) (**C**) and glutathione peroxidase (GP_X_) (**D**) activities. Values are the means ± SD of three independent assays. Values with different superscripts (a–e) are significantly different at *p* < 0.05 as compared to control group.

A large body of evidence shows that oxidative stress induces, with different degrees of importance, protein oxidation, lipids peroxidation and nitrite release, causing accumulation of reactive metabolites which are closely related with gastric ulcer [40]. As shown in Figure 6A, EtOH/HCl- treatment increased the gastric levels of MDA, compared with the normal group (*p* < 0.05). Notably, this increment was prevented in a dose-dependent way by the MMEO pre-treatment (reduction of 41.46%; 51.65% and 69.36% for 250, 500 and 1000 mg/Kg, respectively). Hence, the results suggest that MMEO acts as a ROS scavenger.

SOD, CAT, and GPx are part of the first line of defense against oxidative damage caused by ulcer injuries [41]. Clearly, in ulcerated rats, a decrease in the activity of these enzymes was observed, compared with normal rats, while MMEO (250, 500 and 1000 mg/Kg) and famotidine significantly reversed the ethanol-induced changes in SOD, CAT and GPx levels (Figure 6B–D). As far as we know, there are no previous studies evaluating the potential modulation of Myrtus EO on the activity of SOD, CAT, and GPx. Nevertheless, Porres-Martínez et al. [42] have highlighted the role of α-pinene and 1,8-cineole, major monoterpenes found in *Salvia lavandulifolia* EO as regulators of cellular redox balance.

## 3. Materials and Methods

### 3.1. Plant Material

*M. communis* (Myrtle) leaves were harvested from Errihan Mountain of Seliana governorate. The plant was identified by the botanist of the Biotechnology Center of Borj-Cedria (CBBC), and a voucher specimen [F-RE 27] was deposited at the Herbarium of the Laboratory of Aromatic and Medicinal Plants.

### 3.2. Extraction and Characterization of M. Communis EO

Dried leaves (100 g) were subjected to hydrodistillation for 3 h with 500 mL of distilled water using a Clevenger-type apparatus, according to the European Pharmacopoeia (2016). The obtained essential oil was collected and dried over anhydrous sodium sulfate and stored in sealed glass vials in a refrigerator at 4 °C prior to analysis.

The analysis of the volatile constituents was run on a Hewlett-Packard GC–MS system (GC: 5890-series II; MSD 5972, Palo Alto, CA, USA) equipped by a fused-silica HP-5 MS capillary column (30 m × 0.25 mm ID, film thickness of 0.25 μm). The carrier gas was helium, with a flow rate of 1.2 mL/min. Oven temperature was programmed at 50 °C for 1 min, then 50–280 °C at 5 °C/min and subsequently, held isothermal for 2 min. The injector port and detector were respectively at 250 °C and 280 °C and the split ratio was 1/50. Software adopted to handle mass spectra and chromatograms was a Chem Station. All constituents were identified by comparison of their mass spectra with those in the Wiley 275 GC–MS and FFNSC1.3 libraries and Kovats index. Quantification of *M. communis* EO constituents was determined after normalizing the areas of each detected compounds and expressed as a percentage of total area (%).

### 3.3. Microencapsulation of M. communis Essential Oil and Characterization of the Microspheres

Essential oil from the leaves of *M. communis* (MEO) was added to the maltodextrin, which was previously dissolved in distillated water (ratio 1:1:1) and an emulsion was obtained using a mechanical stirrer operated at 1000 rpm until complete dispersion of the essential oil. Then, mixtures were treated by ultrasonication (480 W, 35 kHz, 100% pulse). The resulting mixtures were spray dried in a Büchi spray dryer (Mini Spray Dryer B-290; Flawil, Switzerland) equipped with 0.7 mm diameter nozzle under an adjusted compressed air pressure of the flow spray at 6 bar and 400 l/h. Outlet and inlet temperatures were maintained at 71 ± 5 °C and 150 ± 3 °C, respectively. Each preparation was collected from the collecting chamber and filled in airtight and placed in hermetic glass bottle and stored at 4 °C for further analysis.

The microencapsulation yield (MEY) was calculated based on the weight of the encapsulants (maltodextrin) and myrtle EO used for the emulsion and on the final weight after drying, according to the following equation:

$$\mathrm{MEY}\;\left(\%\right)=\frac{\mathrm{Weight}\;\mathrm{of}\;\mathrm{the}\;\mathrm{microencapsulated}\;\mathrm{product}\;\mathrm{after}\;\mathrm{spray}\;\mathrm{drying}}{\mathrm{Dry}\;\mathrm{weight}\;\mathrm{of}\;\mathrm{maltodextrin}\;+\;\mathrm{myrtle}\;\mathrm{EO}}$$

The microencapsulation efficiency (ME), i.e., the amount of Myrtus EO retained in the encapsulating matrix, was determined according to Alves et al. [43]. First the total dryer powder was weighed. Then, 10 g of the powder was dissolved in 250 mL of water and transferred to a 500 mL flask. The flask was then attached to the Clevenger apparatus for 3 h of steam distillation. The volume of the EO obtained after the water distillation was multiplied with the density of myrtle EO (0.869 g/mL) to estimate the actual oil content in the capsules. The ME was then calculated according to the following equation:

$$\mathrm{ME}\;\left(\%\right)=\frac{\mathrm{Weight}\;\mathrm{of}\;\mathrm{EO}\;\mathrm{retained}}{\mathrm{Weight}\;\mathrm{of}\;\mathrm{incorporated}\;\mathrm{EO}}$$

### 3.4. Evaluation of the Gastroprotective Effect of MMEO

#### 3.4.1. Animals

Healthy male Wistar rats weighing (≈200 g and 7–9 weeks old) were procured from Tunis Pasteur Institute (B.P. 74.1002 Tunis) and housed in animal cages under standard environmental conditions (temperature 21 ± 1 °C, humidity 60–70%, 12 h light:12 h dark cycle). All animals were fed with standard pellet diet and had free access to drinking water. All experimental procedures were conducted in conformity with institutional guidelines for the care and use of laboratory animals in Tunisia, and the international guidelines on the ethical use of animals (NIH publications No. 80-23). The research was approved by UCSI Ethical Committee ethical code (Ref 202115).

#### 3.4.2. Acute toxicity Test

The safety of oral doses of 250, 500 and 1000 mg/Kg of MMEO was determined by the acute oral toxic test as described by Rujjanawate et al. [44], with slight modifications. The test was based on observational changes among the animals. The control and treated group consisted of six animals each. The control group was treated with saline while the treated groups were given different doses of MMEO. The animals fasted overnight before receiving dosage. Treated groups were administered orally at a dosage of 250, 500 and 1000 mg/kg every day, over 21 days. The animals were observed for any abnormal behavior such as salivation, diarrhea, hyper-excitability, respiratory suffering, and incidence of mortality for the first day, followed by 3, 7, 10, and 21 days after the administration of MMEO, and compared with the control group.

#### 3.4.3. Study Design

HCl/EtOH-induced ulcer model was adopted in the analysis of the gastroprotective effect in rats [44]. The rats were randomly divided into six groups, consisting of six rats per group. The normal control group of rats (group 1) were given only saline solution (10 mL/kg) and were not induced with any drugs. Rats of groups 2 and 6 were given saline solution (10 mL/kg) and famotidine as standard (20 mg/kg), respectively. At 1 h, they were treated with 1 mL/rat of acidified ethanol solution (60 mL EtOH + 1.2 mL HCl + 38.8 mL H_2_O) [45]. Rats in groups 3, 4, and 5 were given MMEO at 250, 500 and 1000 mg/kg, respectively, and induced with the ulcer agent one hour after this pretreatment. All animals were fed by oral gavages with the help of a feeding tube (16 G) in oral administration. After 1 h, the stomachs of the sacrificed rats in CO_2_ chamber were opened along the greater curvature and washed with water and assessed for the evaluation of gastric mucosal damage [44].

#### 3.4.4. Histopathology

For histopathological examinations, stomachs were fixed in 3% formaldehyde, then cut in pieces, dehydrated in graded ethanol, embedded in paraffin blocks and finally cut in 5 μm sections. Sections were then stained with hematoxylin and eosin (H&E) solutions. The specimens were conducted under light microscope.

#### 3.4.5. Evaluation of Gastric Mucosal Damage

The lesions and hemorrhagic erosions in the gastric mucosa were examined microscopically. The different parameters were calculated as follows:

Ulcer index (UI) = (Average number of severity score) × (% of rats with ulcers number of animals);

The numbers of severity score for irritation and for ulcers were measured according to Lwoff (1971) following 5-point scale: 0 = neither ulcer nor irritation; 1 = irritation; 2 = 1 or 2 ulcers; 3 = 3 or 4 ulcers; 4 = >4 ulcers;

Ulceration percentage (%UP) = (UI × 100)/3;

Degree of protection (DP) = PU (control group)−PU (treated group with extract or famotidine);

Healing percentage (% HP) = (UI (control group)−UI (treated group with extract or famotidine) × 100)/UI (control group).

#### 3.4.6. Evaluation of Gastric Secretions

Pylorus ligation method was used to study the gastric secretions [46]. Concentrations of 0, 250, 500 or 1000 mg/kg of MMEO or famotidine were administered orally to 24-h fasted rats, with free access to water except for the last hour before pyloric ligation. The abdomen of anesthetized animals was opened below the xiphoid process and the pylorus portion of the stomach was uplifted and ligated avoiding any traction to the pylorus or damage to blood supply. The stomach was then returned in the abdomen and the incision was sutured by interrupted sutures. The rats were sacrificed 4 h later by an overdose of ether. The stomach was removed and its contents subject to measurement of gastric pH and fluid volume (mL).

#### 3.4.7. Biochemical Analysis

Gastric tissue homogenate was freshly prepared in phosphate buffer 100 mM (pH 7) containing a mixture of mammalian protease inhibitors and then centrifuged at 3000× *g* for 10 min (4 °C). The supernatant was used for the measurement of nitric oxide (NO) levels using Griess reagent [47], and the monitoring of enzymatic activities. The activity of superoxide dismutase (SOD) was determined by a modified epinephrine assay [48], while Catalase (CAT) and glutathione peroxidase (GPx) activities were estimated by screening the H_2_O_2_ consumption, following the procedures of Aebi and Rtibi et al. [48,49], respectively. For lipid peroxidation estimation, gastric mucosa homogenates were blended in BHT-trichloroacetic acid (TCA) solution (1% BHT (*w*/*v*) dissolved in 20% TCA (*w*/*v*)) and then the homogenate was centrifuged at 1000× *g* for 5 min at 4 °C, followed by the monitoring of the levels of MDA of the supernatant, according to the method of Rtibi et al. [49].

### 3.5. Statistical Analysis

Data were analyzed using one-way analysis of variance using Graph Pad Prism, version 6. Means were compared according to Tukey’s test at *p* < 0.05 when significant differences were found. Multivariate data analysis was carried out using principal component analysis (PCA). The PCA type used was Pearson’s correlation and it was done using XLSTAT, considering variables centered on their means and normalized with a standard deviation of 1.

## 4. Conclusions

In conclusion, the herein gathered results show that the treatment of rats with MMEO significantly protected the gastric mucosa from lesions caused by ethanol/HCl. Similarly, oral administration of MMEO at 250–1000 mg/kg did not show any signs of toxicity in animals, as evaluated by macroscopic and microscopic changes in the stomach. The data provides evidence to suggest that encapsulated MEO has a potential applicability in the treatment of acute gastric ulcers. More importantly, treatment of animals with MMEO successfully inhibited oxidative damage and reversed the impairment of the antioxidant system in the intestinal mucosa.

## Figures and Tables

**Table 1:** Chemical composition of Myrtus communis essential oil.

Compounds	Retention Time (mn)	Relative Percentage (%)
α-pinene	5.85	11.10
Limonene	8.10	1.63
1,8-Cineole	8.18	9.98
linalool oxide	9.25	0.38
α-Terpinolene	9.67	0.46
Linalool	9.94	14.92
α-Terpineol	12.49	4.64
Linalyl acetate	14.2	4.61
Myrtenyl acetate	16.13	30.59
Camphene	16.74	0.83
Neryl acetate	17.07	0.38
Geranyl acetate	17.57	1.62
Methyleugenol	18.13	2.51
α-Humulene	19.49	0.77

**Table 2:** Effect of oral pre-treatment with microencapsulated myrtle essential oil (MMEO) on gastric ulcer parameters in rats (*n* = 6/group).

	Group 1 (Normal Control)	Group 2 (Ulcer Control)	Group 3 (MMEO 250 mg/kg)	Group 4 (MMEO 500 mg/kg)	Group 5 (MMEO 1000 mg/kg)	Group 6 (Famotidine) (20 mg/Kg)
UI	-	2.66 ^a^	1.87 ^b^	1.72 ^c^	1.63 ^d^	1.50 ^e^
UP	-	85.16 ^a^	66.5 ^b^	22 ^c^	19.66 ^d^	22.83 ^c^
PP	-	-	26.3 ^c^	83.16 ^a^	83.33 ^a^	69.65 ^b^
HP	-	-	59.11 ^c^	65.23 ^b^	69.97 ^a^	43.67 ^d^
GpH	5.09 ^a^	2.04 ^e^	2.4 ^d^	2.6 ^c^	3.48 ^b^	3.02 ^c^
GV	4.26 ^a^	1.75 ^e^	2.75 ^d^	3.78 ^b^	3.63 ^c^	3.80 ^b^

UI: Ulcer index (mm); GV: gastric volume ml; GpH: gastric pH; UP: ulceration percentage, PP: percentage of protection; HP: healing percentage. Group 1: Normal control (10 mL/kg of NaCl 0.9%); Group 2: Ulcer control (pre-treated with 10 mL/kg of NaCl 0.9% followed by EtOH/HCl); Groups 3, 4, 5: pre-treated with MMEO at 250, 500 and 1000 mg/kg, respectively, followed by EtOH/HCl; Group 6: Positive control (pre-treated with famotidine 20 mg/kg, followed by EtOH/HCl). Values are the means of three replicates and standard deviation. Values within the same line with different superscripts (a, b, c, d, e) are significantly different at *p* < 0.05.

**Table 3:** Pearson’s correlation coefficients of ulcer-protective parameters with EO components.

Variables	PP	UP	HP
PP	1	−0.9524	0.9352
UP	−0.9524	1	−0.7828
HP	0.9352	−0.7828	1
α-Pinene	0.6805	−0.6083	0.5260
Limonene	0.2293	−0.3642	0.0441
1,8-Cineole	0.1698	−0.1436	0.1777
Linalool oxide	−0.0811	−0.0758	−0.2473
α-Terpinolene	−0.2017	0.0944	−0.3026
Linalool	−0.0164	0.0689	0.0501
α-Terpineol	−0.0779	0.1274	−0.0133
Myrtenol	−0.1339	0.1054	−0.1496

UP: Ulceration percentage; PP: percentage of protection; HP: healing percentage.

## Data Availability

Data is contained within the article.

## Sample Availability

Samples of the compounds are available from the authors.
